# Comparison of SHD-IBG and PVIBGT in ONFH including mechanical and pathological analysis of failure cases

**DOI:** 10.1038/s41598-024-65197-9

**Published:** 2024-06-22

**Authors:** Jiahao Sun, Bowen Ma, Zhiyuan Chen, Tianwei Xia, Jirong Shen

**Affiliations:** 1 Nanjing University of Chinese Medicine, Nanjing, Jiangsu Province China; 2 Jiangsu Province Hospital of Chinese Medicine, Affiliated Hospital of Nanjing University of Chinese Medicine, Nanjing, Jiangsu Province China

**Keywords:** Osteonecrosis of the femoral head, Pedicled vascularised iliac bone graft transfer, Surgical hip dislocation, Impacting bone grafts, DCE-MRI, Hip-preserving, Computational biology and bioinformatics, Risk factors, Preclinical research

## Abstract

Currently, there is a lack of relevant research on the efficacy difference between SHD combined with IBG and PVIBGT in the treatment of osteonecrosis of the femoral head(ONFH). Firstly, this study intends to compare the effectiveness of surgical hip dislocation combined with impacting bone grafts (SHD-IBG) and pedicled vascularised iliac bone graft transfer (PVIBGT) in treating ONFH. And the study investigates patients who suffered from hip preservation failures from both groups to better comprehend failure reasons. 30 patients (34 hips) with ARCO stage IIIA femoral head necrosis were selected between January 2012 and July 2022. They were divided into group A(SHD-IBG) and group B (PVIBGT) according to different surgical methods. Firstly, compared the 1-year effect between SHD-IBG and PVIBGT at 1 year postoperatively; Secondly, assessed the medium and long-term efficacy of SHD-IBG hip preservation treatment; Lastly, based on study of the femoral head removed from patients with hip preservation failure in the two groups, the reasons for the failure of hip preservation were comprehensively analyzed in the two groups. Group A: 11 males (13 hips), 4 females (4 hips);Group B: 9 males (11 hips), 6 females (6 hips).Firstly, the average Harris scores of the two groups at 1 year after surgery: preoperative: 70.7, 1 year after surgery: 78.9 in group A; preoperative: 69.5, 1 year after surgery: 81.5 in group B. The differences were statistically significant (P < 0.05).Compared to the preoperative period, quantitative analysis by DCE-MRI showed an increase in perfusion in the necroticarea and an improvement in hyperperfusion in the repair-responsive area one year after the surgery. Secondly, in group A, the hip preservation rate was 88.2% at 2.5–11 (average of 77 months) years of follow-up, and the mean Harris score at the last follow-up was 73.2.Semi-quantitative analysis of postoperative DCE-MRI showed that the perfusion curves of necrotic and repaired areas were similar to those of the normal area. This suggests the instability within the femoral head had been effectively improved, and the perfusion had partially recovered. Thirdly, according to Micro-CT and pathologica studies of patients with hip preservation failure in these two groups, all these patients' femoral head was significantly collapsed and deformed. Their trabeculae was thin and partially disorganized, with fractures in the subchondral bone and separation of the cartilage from the subchondral bone. The necrotic areas had sparse trabeculae, disorganized arrangement, loss of continuity, and disappearance of cells in the trabecular traps. The necrotic area was covered with fibrous tissue, and partial restoration was observed in the repair area. Mechanical finite element analysis showed that the maximum equivalent force was observed in the weight- bearing area and the cortical bone surrounding the shaft of femurand. The result of DCE-MRI showed that the repair reaction area exhibited abnormal hyperperfusion. In this study, the efficacy of SHD-IBG and PVIBGT was compared at 1 year after operation, and the long-term follow-up of SHD-IBG was 2.5–11 (mean 77 months) years, combined with DCE-MRI results, we found that the short-term effect of PVIBGT was more significant than that of SHD-IBG. SHD-IBG can achieve satisfactory hip preservation in the medium and long term follow-up.

## Introduction

Osteonecrosis of the femoral head (ONFH) is a highly morbid and disabling arthropathy, prevalent in young and middle-aged populations, and associated with various etiological elements(such as hormone use, alcohol abuse, and hip trauma)^1^.

In 2001, Ganz et al.^2^ first proposed the technique of SHD of the hip joint, and then, with the widespread use of IBG in hip preservation surgeries, the SHD-IBG also came into being and was used for ONFH preservation treatment^3^. In recent years, PVIBGT has become an important procedure for the treatment of end-stage ONFH with a large necrotic range^4^.

The present study retrospectively analysed the clinical data of 30 patients (34 hips) with ONFH who were treated with SHD -IBG or PVIBGT from June 2012 to January 2023. Firstly, because the two surgical procedures were carried out at different times in our hospitals, we analysed and compared the efficacy of the two different surgical procedures in the treatment of ONFH at 1 year postoperatively. patients with SHD-IBG were followed up for an average of 77 months postoperatively to analyse their long-term efficacy in treating ONFH. Secondly, the effects of the two surgical procedures on the recovery of femoral blood supply were evaluated based on Dynamic contrast-enhanced magnetic resonance imaging (DCE-MRI)^5^. Finally, a comprehensive analysis of the two groups of patients with hip preservation failure was carried out by combining mechanical analysis, Micro-CT and pathological studies to summarise the factors leading to hip preservation failure.

## Methods and basic clinical information

### Patient selection criteria

A total of 30 patients (all patients with preoperative femoral head necrosis stage are ARCO IIIA) were included from 01/2012 to 01/2023, and the 30 patients were divided into two groups, A and B, according to the different surgical methods. Among them, 15 patients (17 hips) SHD-IBG in the test group (Group A) and 15 patients (17 hips) in the control group in PVIBGT (Group B).

### Ethical approval

This prospective, randomised controlled study was approved by the Medical Ethics Committee of the Affiliated Hospital of Nanjing University of Traditional Chinese Medicine and was registered with the China Clinical Trial Registry (ChiCTR1900024981). After providing all the enrolled patients with detailed information about the surgical procedure, their written informed consent was obtained. All procedures are carried out in accordance with the “Helsinki Declaration” of the World Medical Association.

The date of the first trial registration is 06/08/2019 and registration number is ChiCTR1900024981.

### Surgical methods

Surgery was performed by the same physician in both groups.

**Figure 1:**
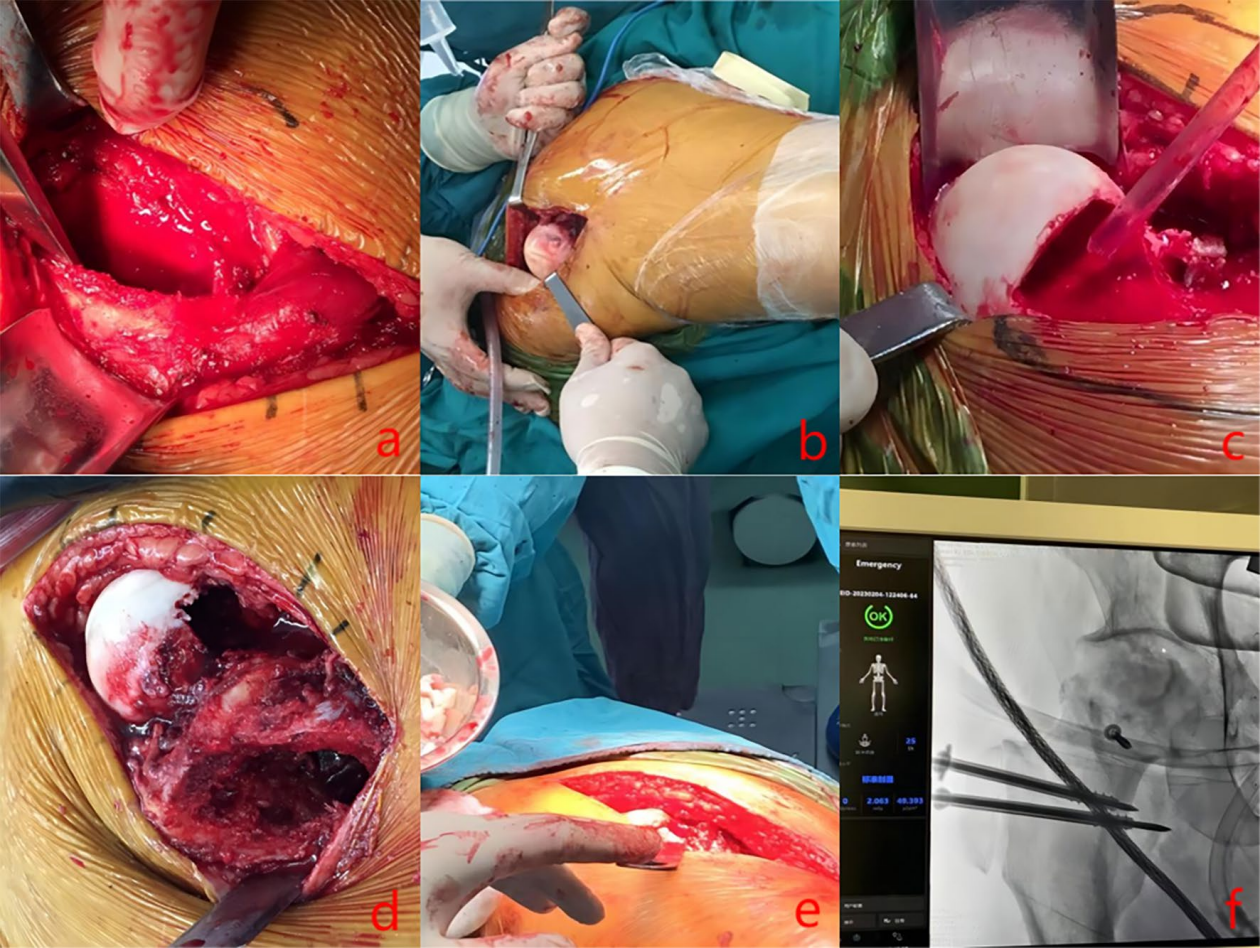
(**a**, **b**) opening the joint capsule, dislocating the hip joint and exposing the femoral head; (**c**) cleaning the dead bone in the dead bone area until the femoral head is well oozing blood and protecting the cartilage; (**d**, **e**) taking the cancellous bone around the ilium, impacting with allogenic cancellous bone before opening and fixing the hip head; (**f**) fixed bone graft with cannulated screw in the greater trochanter, and reduced the femoral head.

Group A: (1) Surgical dislocation of the hip joint: the patient was placed in the lateral position after general anesthesia, and a longitudinal incision of about 10 cm in length was made on the lateral side of the hip joint centered on the apex of the greater trochanter. The skin, superficial fascia, deep fascia, and tensor fascia lata were incised and entered from the anterior edge of the gluteus maximus. The tensor fascia lata was pulled forward and the gluteus maximus was pulled backward. The hip was rotated slightly, and the vastus lateralis muscle was separated to the distal femur along the posterosuperior margin of the greater trochanter (gluteus medius insertion) and the posterior margin of the femoral shaft (vastus lateralis insertion). A pendulum saw was used to cut the greater trochanter from behind to forward along this line, and the thickness of the osteotomy was 1.0–1.5 cm. Adhere to the forward traction sections of bone and the vastus lateralis muscle, complete protection extorsion muscle group. The Hohmann retractor was inserted into the osteotomy gap, and the hip joint was blunt-separated along the tip of the greater trochanter at flexion, abduction, and external rotation positions. The joint capsule was exposed and sliced in a Z-shape. A single hook was pulled from the joint around the base of the femoral neck outward, and a curved shear was used to enter the acetabulum and cut the round ligament, so that the femoral head was dislocated outward. (2) After surgical dislocation of the hip, a hole in the femoral head was drilled with 1.5 mm Kirschner wire, a window was opened in the head and neck, a grinding drill was used to remove the sclerosed bone, and a curette was used to remove a large amount of dead bone in the head, partially to the subchondral bone of the femoral head. (3) An oblique incision of about 5 cm in length was made at the left anterior superior iliac spine, subcutaneous tissue was cut to obtain a cortical bone block of about 3 cm in length, and cancellous bone was taken from the osteotomy of the greater trochanter and the ilium. (4) At the opening of the femoral head and neck, the removed iliac bone was placed along the direction of the lateral column and pressed firmly. First, a Kirschner wire was temporarily fixed, and then a hollow screw was fixed after an electric drill was drilled. Will remove the cancellous bone implant and, until the normal spherical femoral head. The femoral head was reduced and the joint capsule was sutured. (5) The osteotomy of the greater trochanter was temporarily fixed with towel forceps + 2 Kirschner wires, and then fixed with 2 hollow screws (A0) after electric drilling. See Fig. 1 for details.

**Figure 2:**
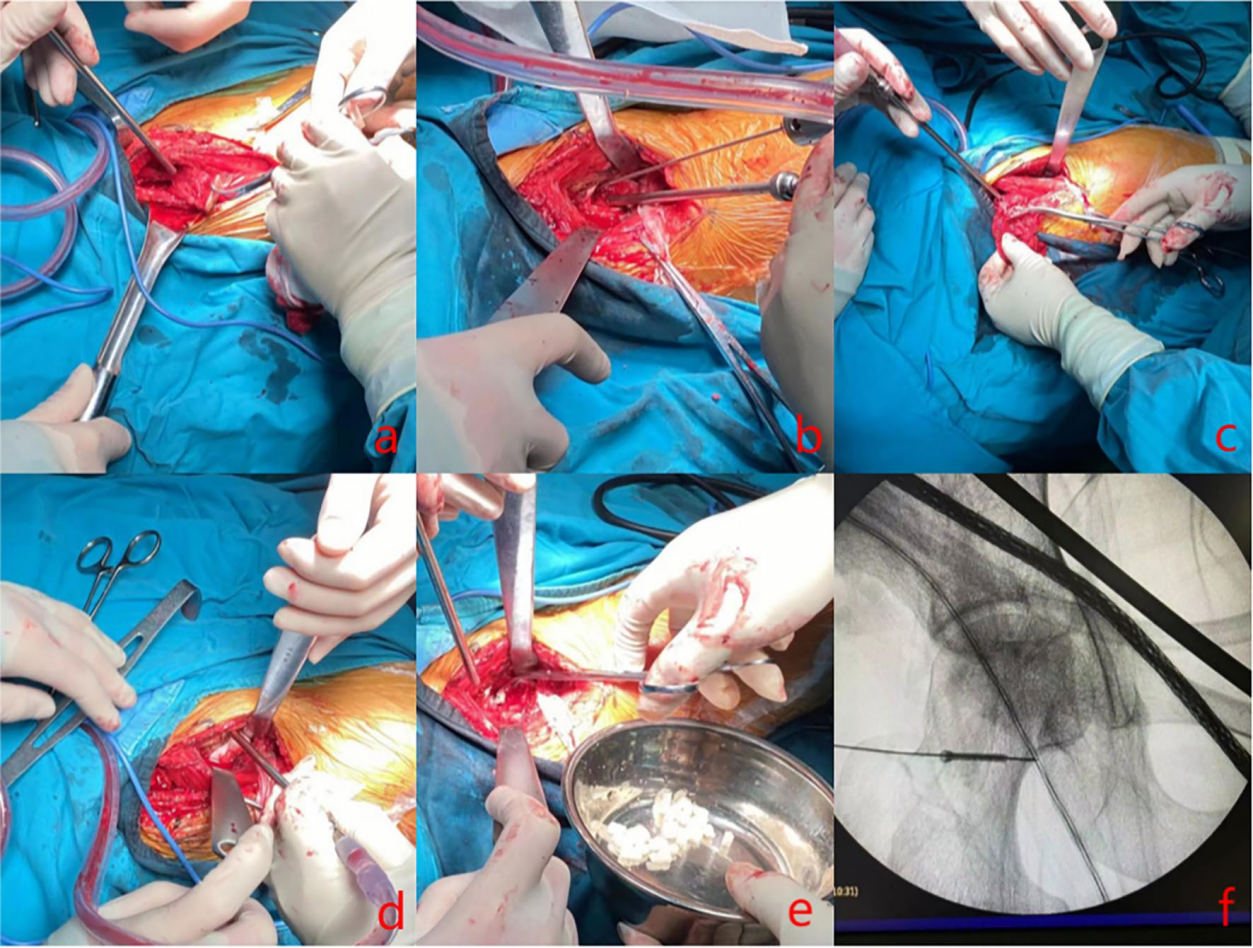
(**a**) taking the iliac bone graft and excavating the cancellous bone; (**b**) exposing the femoral head; (**c**) removing the sclerotic and necrotic bone by high-speed abrasive drilling; (**d**) filling the necrotic area with cancellous bone and filling it up with compression to hold up the collapsed femoral head; (**e**) filling the necrotic area further with cancellous bone of femoral flap which is rich in blood supply to hold up the anterior lateral column; (**f**) securing the musculoskeletal flap obliquely downward from the top by screwing the screws and screwing the screws completely into the femoral head.

Group B: (1) patients after general anesthesia lie on your back, side hip pad high 30°. The surgical position was chosen on the line connecting the anterior superior iliac spine and the outer edge of the patella, along the ilium to 3 cm below the anterior superior iliac spine toward the lateral aspect of the greater trochanter. Then, it was turned back to the connecting line to form a nearly "S"-shaping incision, which was about 8–12 cm long. (2) The skin and subcutaneous tissue were cut in turn to protect the lateral femoral cutaneous nerve. One retractor was used to pull the sartorius and rectus femoris inward, and the other retractor was used to open the tensor fasciae externally to expose Huter's space. From the lower end of the Huter's hiatus, the ascending branch of the lateral circumflex femoral artery was located layer by layer and protected (this vessel supplies blood to the iliac flap along with the vastus tensor fasciae latae), and from the branch of the lateral circumflex femoral artery, part of the vastus tensor fasciae latae muscle was incised from the bottom up, near the starting point of the lateral circumflex femoral artery, until the vastus tensoriae latae muscle was located near the starting point of the anterior superior iliac spine. (3) The iliac bone block of the anterior superior iliac spine with the origin of the tensor fascia lata (with the free tensor fascia lata with the ascending branch of the lateral circumflex femoral artery) was taken, the size of the bone block was about 3 × 3 × 1 cm, and the cancellous bone of the iliac bone was harvested for intraoperative bone grafting. (4) The anterior hip joint capsule was incised to expose the femoral head, and then the collapsed cartilage was removed with a bone knife to expose the subchondral necrotic bone. (5) With high-speed grinding drill to remove hardened bone, and then with a curet thoroughly remove necrotic bone and hardening of the bone. (6) The necrotic area was filled with cancellous bone taken from the iliac bone and compressed with an impaction rod to prop up the collapsed femoral head. (7) The cancellous bone surface of the musculocutaneous bone flap with rich blood supply was oriented towards the femoral head to further fill the necrotic area, the anterolateral column was proheld up, and the iliac bone flap was transferred to the fenestration of the femoral head. (8) The iliac bone flap was temporarily fixed with Kirschner wire, and the titanium nail with the appropriate length was selected after the depth sounder measurement. Then the titanium nail was screwed in with a screwdriver to fix the bone flap. See Fig. 2 for details.

### Postoperative treatment and postoperative follow up

The patients were followed up at 1, 3, 6, 12 months and every 6 months after the operation. At the follow-up, orthopantomograms and frog X-rays of the hip joint and CT examination of the hip joint were performed to observe whether there was any collapse of the femoral head and the healing of the bone block; the Harris score was used to assess the function of the hip joint.

DCE-MRI was used to observe the blood supply around the femoral head, and the region of interest (ROI) was drawn in the necrotic area, repair area, and the greater trochanter of the femoral head, and the time-intensity curve (TIC) was obtained for all the pixel points in the region of interest. The area under the curve (IAUGC), the contrast-enhanced signal peak (CER), the maximum slope of the curve (MaxSlope), the volume transfer constant (Ktrans), the volume rate constant (Kep), and the volume fraction of extravascular extracellular space (Ve) were calculated.

### Analysis of patients with hip preservation failure

#### Mechanical analysis

In this study, Mimics Research 21.0, 3-matic Research 13.0, Geomagic and Abaqus finite element software were used to model and mechanically analyse the removed femoral head.

#### Micro-CT analysis

The removed femoral head was separated from the surrounding muscles, tissues and ligaments and the femoral head sample was scanned using a small animal in vivo micro-CT imaging system Quantum GX2 (PerkinElmer, USA).

#### Pathological studies

After decalcification of the removed femoral head, the sections were stained with HE.

### Statistical analysis

SPSS 27.0 software was used to analyse the data. The data were expressed as ${\bar{\text{x}}} \pm {\text{s}}$. After the normality test and the chi-square test, the independent samples *t*-test or non-parametric test was used for the enumeration data, and the chi-square test was used for the count data. The differences were considered significant at *P* < 0.05. The test level of *α* was 0.05.

### Informed consent

Informed consent was obtained from all individual participants included in the study.

## Results

### Patient information

Group A: male, 11 patients (13 hips), female 4 cases (4 hips); An average age of 32. There were 4 cases (5 hips) of steroid-induced ONFH, 4 cases (5 hips) of alcohol-induced ONFH, 1 case (hip) of traumatic ONFH, and 6 cases (6 hips) of other ONFH.

Group B: 9 cases (11 hips) male, female 6 cases (6 hips); An average age of 30.5 years. There were 8 cases (9 hips) of steroid-induced ONFH, 1 case (2 hips) of alcohol-induced ONFH, 1 case (1 hip) of traumatic ONFH, and 5 cases (5 hips) of other ONFH.

There was no significant difference in gender, BMI, age, operation time, average hospital stay, and type of ONFH between the two groups (P > 0.05). See Table 1 for details.

**Table 1:** Comparison of clinical data of two groups of patients.

Patient information	Group A (n = 15)	Group B (n = 15)
Sex (m/f)	11/4	9/6
BMI(kg/m^2^)	21.6	20.7
Average age (years)	32	30.5
Average operating time (min)	143	214
Average length of stay (day)	14	16
Type of necrosis (hormonal/alcoholic/traumatic/other)	4/4/1/6	8/1/1/5

There were no statistic differences in patients' gender, age, course of disease, sides, types, and stages of ONFH (*P* > 0.05).

### Comparison of the two groups of patients at 1 year postoperatively

#### Harris score

**Figure 3:**
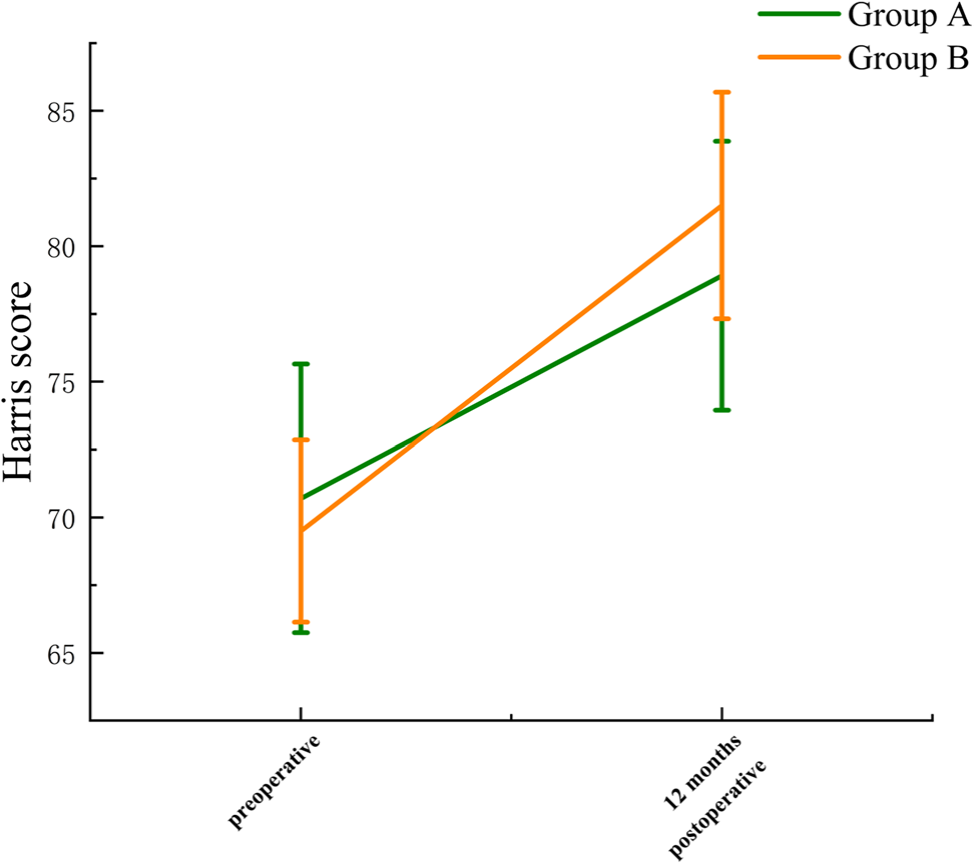
Change in mean Harris score preoperatively and 12 months postoperatively in both groups of patients. The average Harris scores of both groups at 1 year after surgery were significantly higher than those before surgery and the differences were statistically significant (P < 0.05).

The average Harris scores of both groups at 1 year after surgery were significantly higher than those before surgery. The average Harris score of Group A was 70.7 before surgery and 78.9 at 1 year after surgery. The average Harris score of Group B was 69.5 before surgery and 81.5 at 1 year after surgery, and the differences were statistically significant (P < 0.05) (Fig. 3).

#### Imaging data

**Figure 4:**
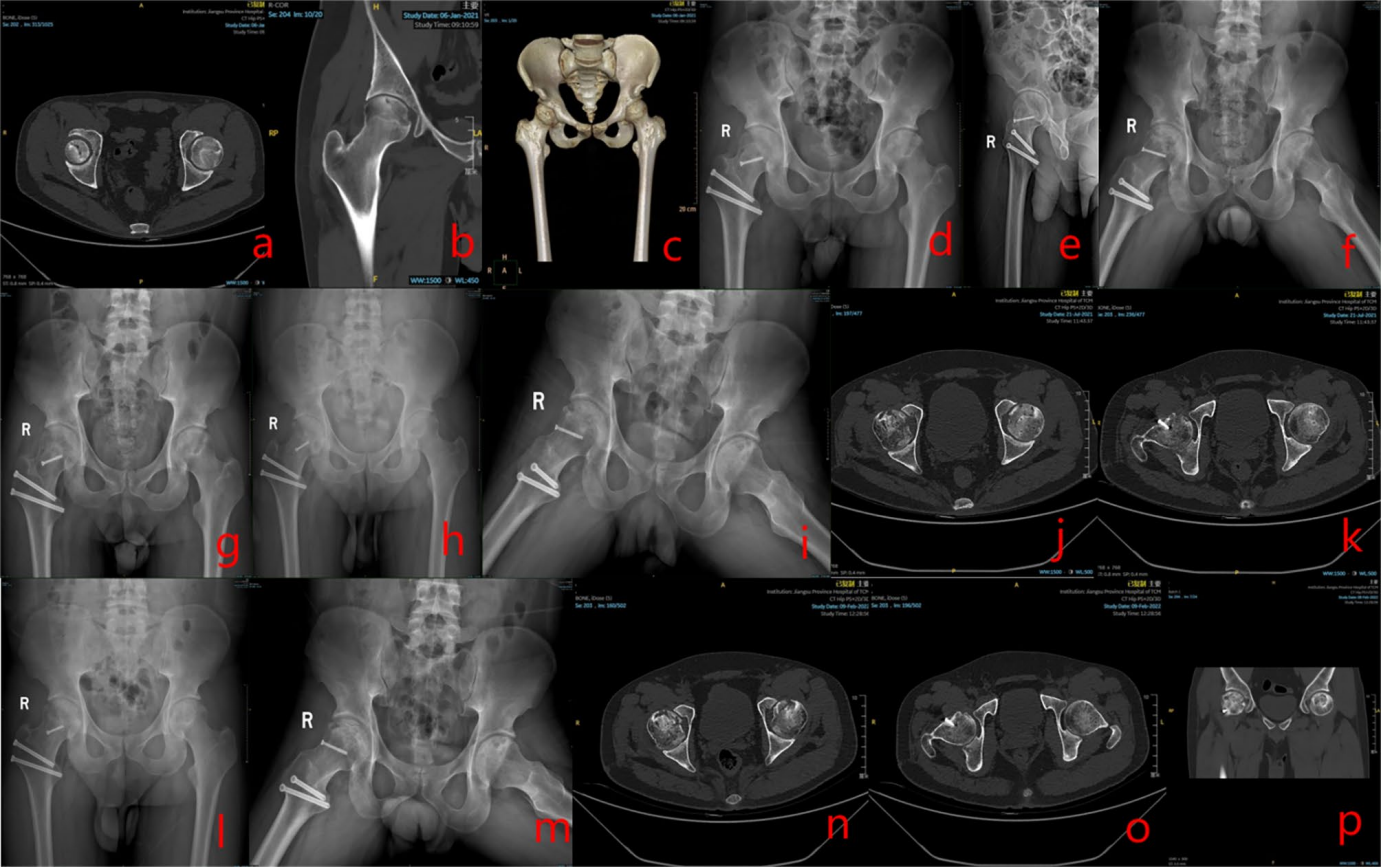
Preoperative (**a**–**c**), postoperative (**d**–**e**), 3 months postoperative (**f**–**g**), 6 months postoperative (**h**–**k**), and 12 months postoperative (**l**–**p**) X-rays, CT and 3D reconstruction.

Group A: Yin XX, male, 23 years old, bilateral SONFH, right hip SHD-IBG performed. See Fig. 4 for details.

**Figure 5:**
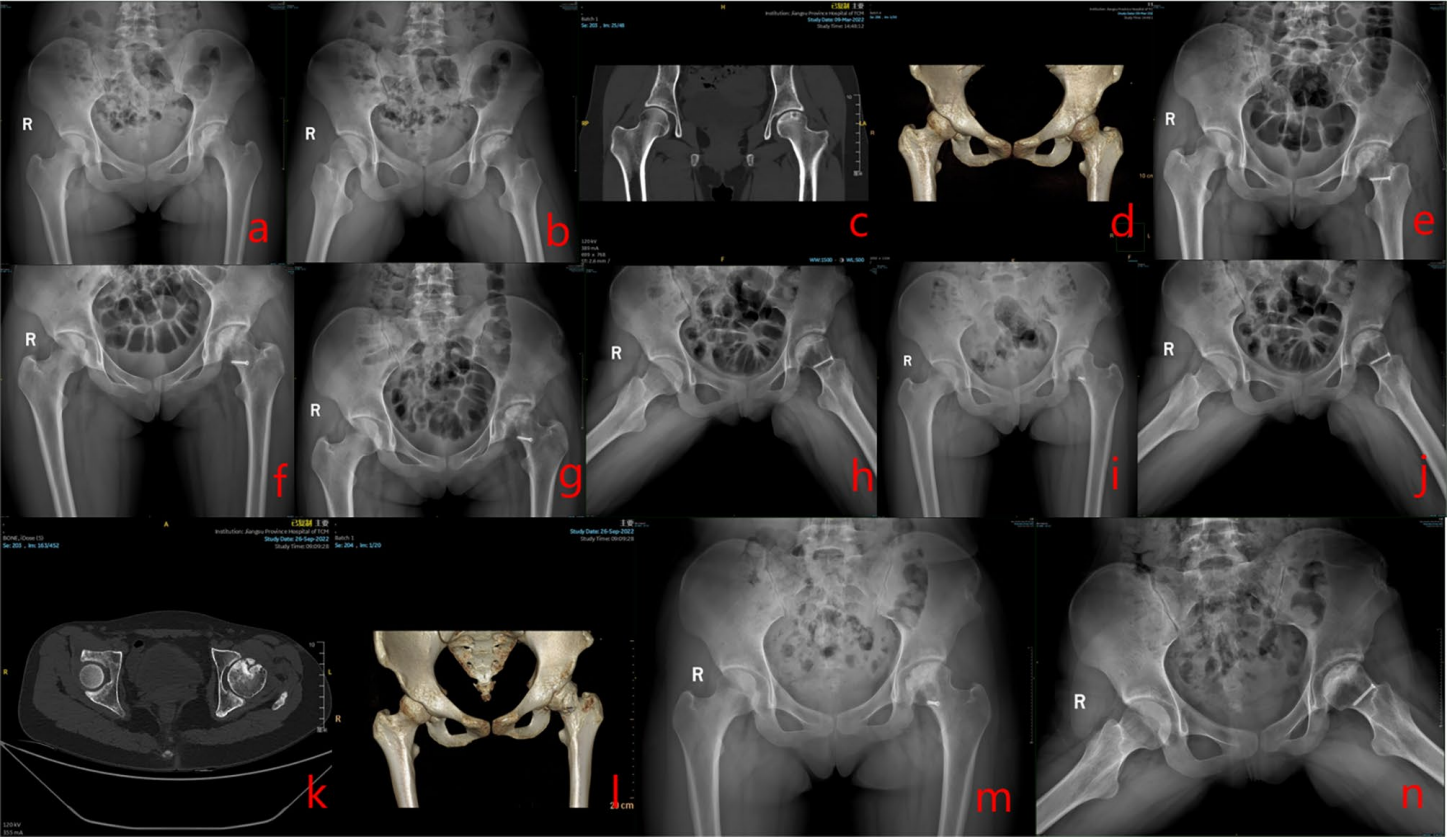
Preoperative (**a**–**d**), postoperative (**e**), postoperative 1 month (**f**), 3 months (**g**–**h**), 6 months (**i**–**j**) and postoperative 12 months (**k**–**n**) X-rays, CT and 3D reconstruction of a typical patient in group B.

Group B: Zhang XX, female, 18 years old, left SONFH, left hip PVIBGT. See Fig. 5 for details.

### Semi-quantitative and quantitative analysis of DCE-MRI

#### Semi-quantitative analysis of typical DCE-MRI in two groups of patients

**Figure 6:**
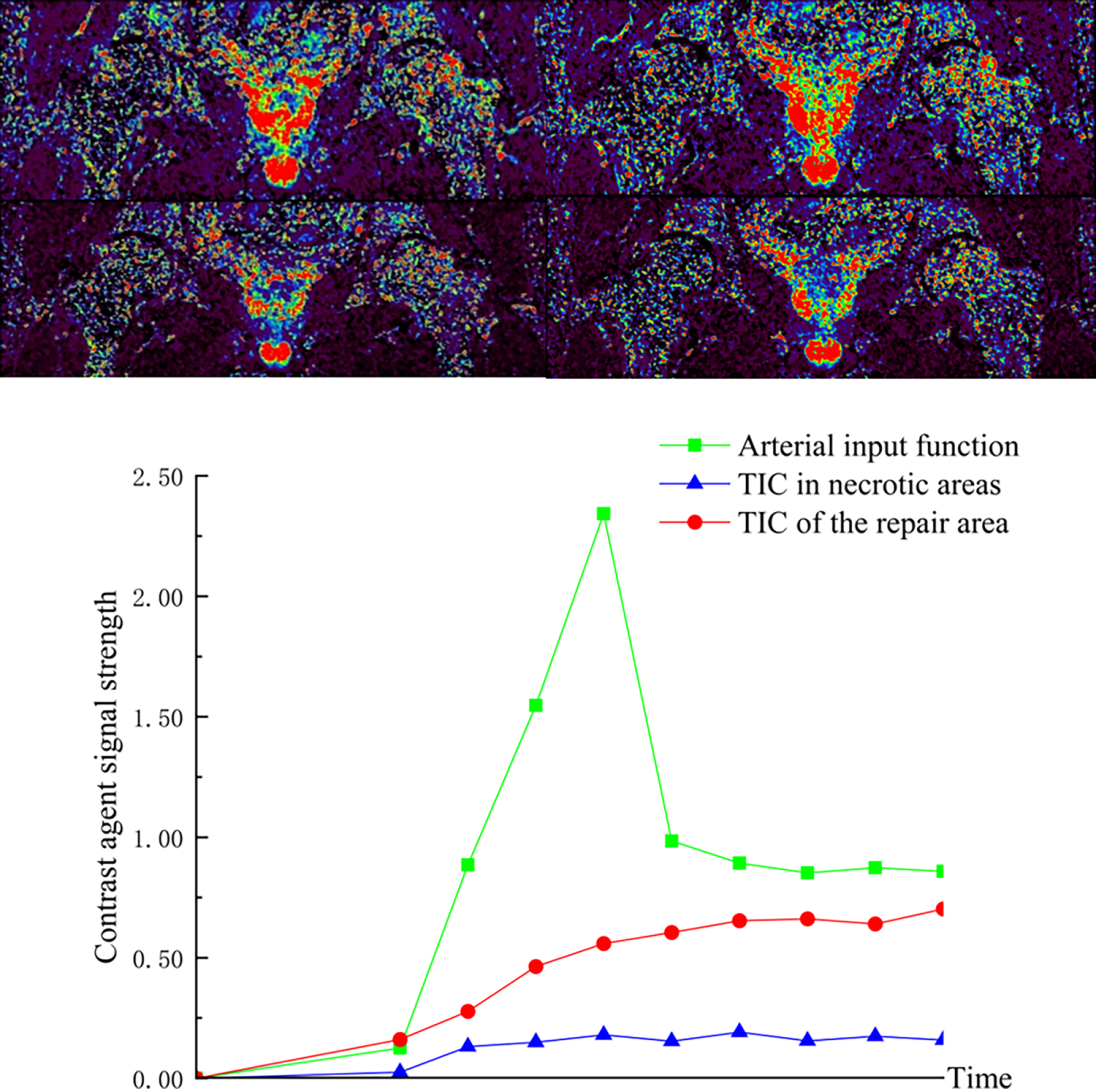
IAUGC and Ktrans synthetic pseudo-colour image and time intensity curve (TIC).

Group A: Du XX, male, 27 years old, right SONFH, right hip SHD-IBG, 5 years postoperative dynamic enhanced nuclear magnetic evaluation of blood supply. See Fig. 6 for details.

**Figure 7:**
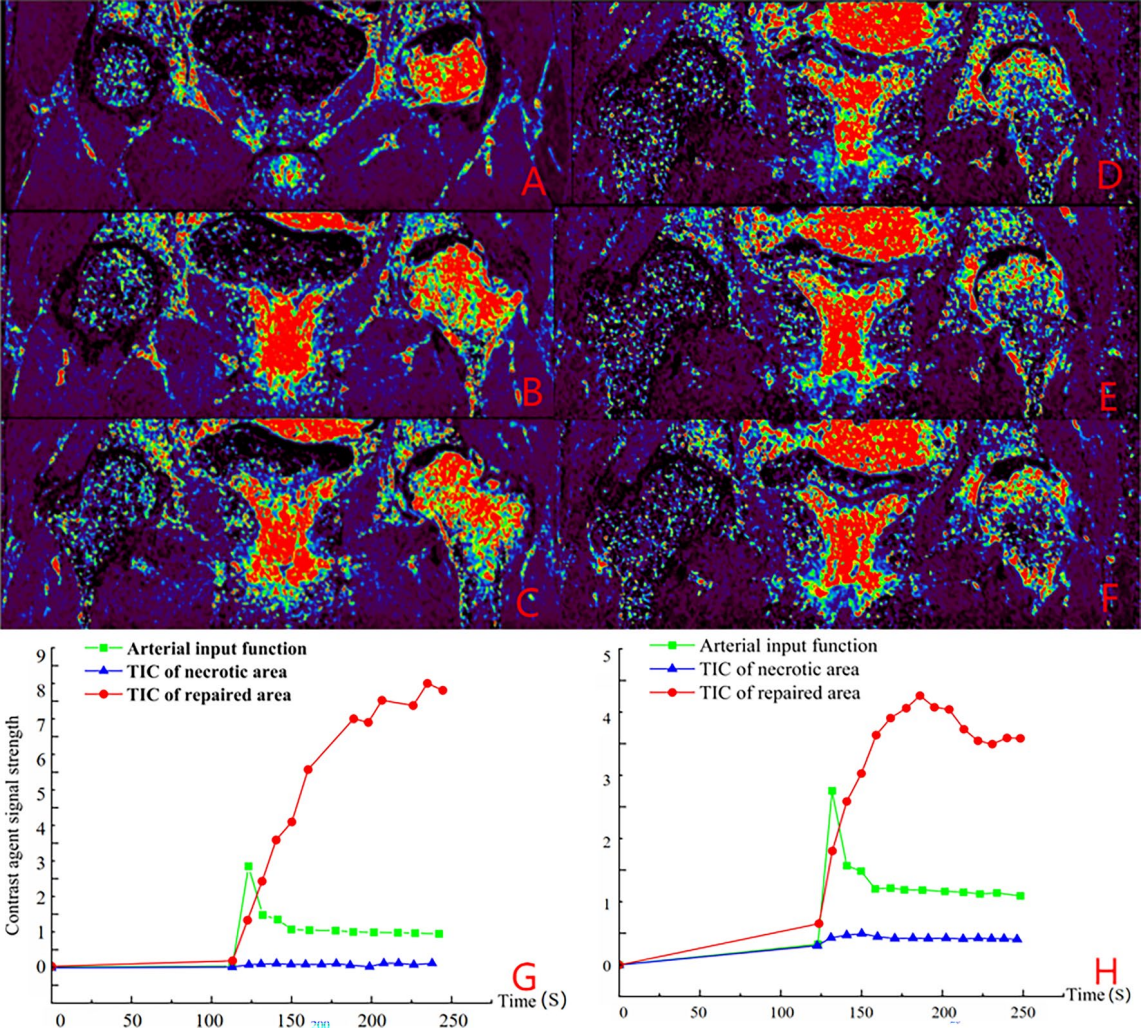
IAUGC and Ktrans synthetic pseudo-colour images and time intensity curves (TIC). Figures (**A**, **B**, **C**, **G**) are preoperative and Figures (**D**, **E**, **F**, **H**) are 12 months postoperative. Comparison of the two figures shows that perfusion increased in the necrotic area and peak of perfusion decreased in the repaired area at 12 months post-operation.

Group B: Zhang XX, female, 18 years old, left SONFH, performed left hip PVIBGT. See Fig. 7 for details.

#### Comparison of changes in quantitative analysis of DCE-MRI between preoperative and 1 year postoperative in group B patients

**Figure 8:**
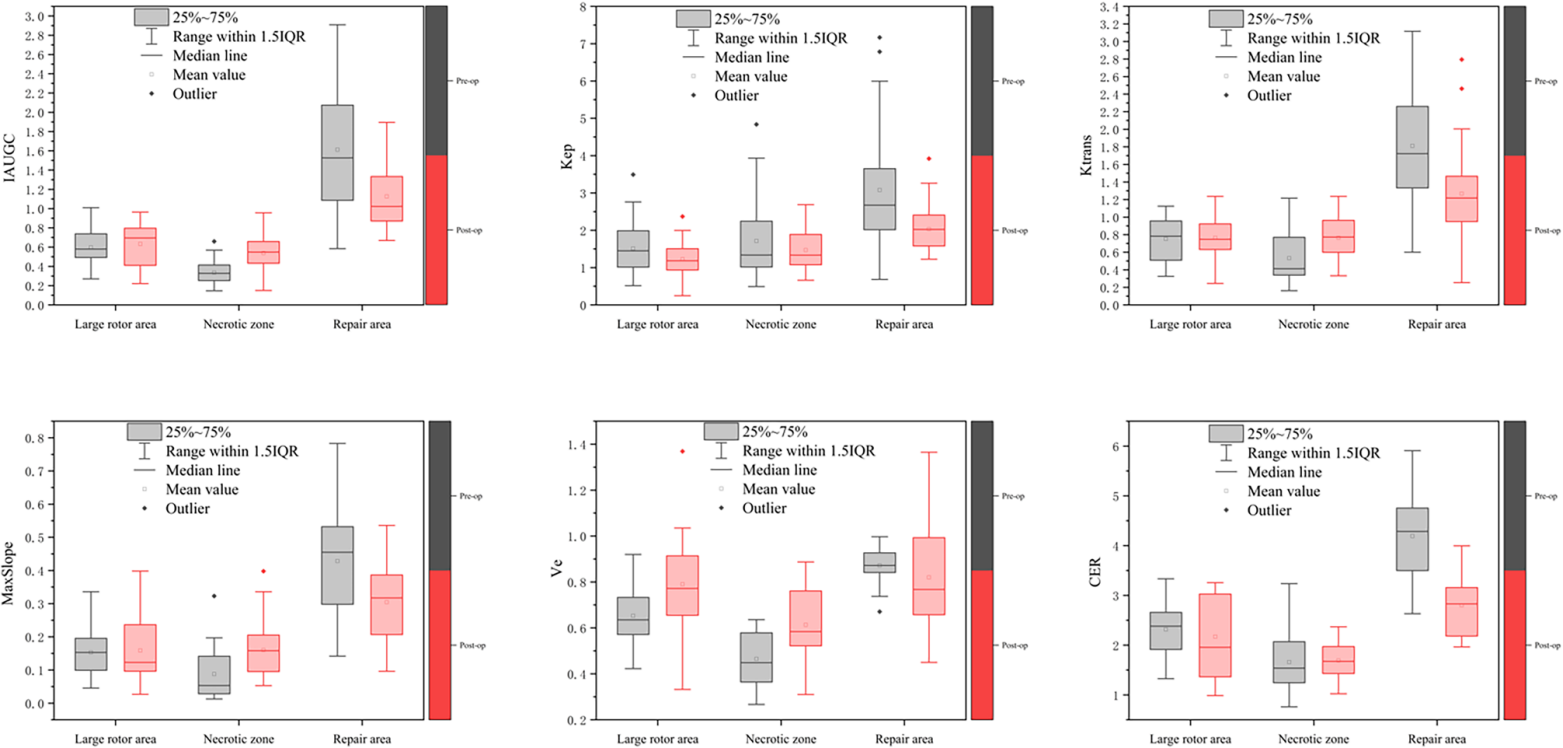
Comparing the perfusion parameters of different regions before and after surgery, the IAUGC, KTrans, MaxSlope, and Ve in the necrotic area after 1 year were all better than those before surgery, with significant differences (P < 0.05), while there was no significant difference in CER and Kep (P > 0.05); The IAUGC, Kep, KTrans, MaxSlope, and CER in the repair response area were significantly lower than those before surgery one year after surgery (P < 0.05), while there was no significant difference in Ve (P > 0.05).

See Fig. 8 for details.

### Results of average of 77 months of follow-up of patients in group A

#### Change in Harris score

**Figure 9:**
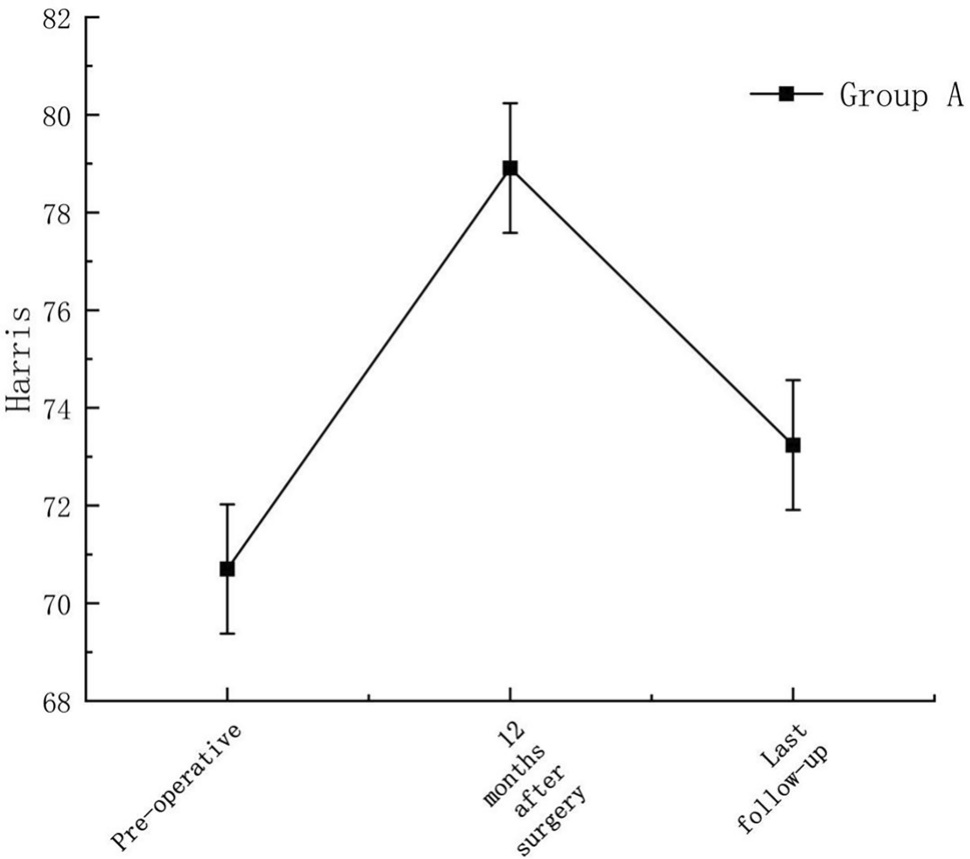
Change in Harris score in group A patients.

By following up Group A for 2.5–11 (mean 77 months) years, the hip preservation rate was: 88.2%, and the mean Harris score at final follow-up was: 73.2. See Fig. 9 for details.

#### Survival curve

**Figure 10:**
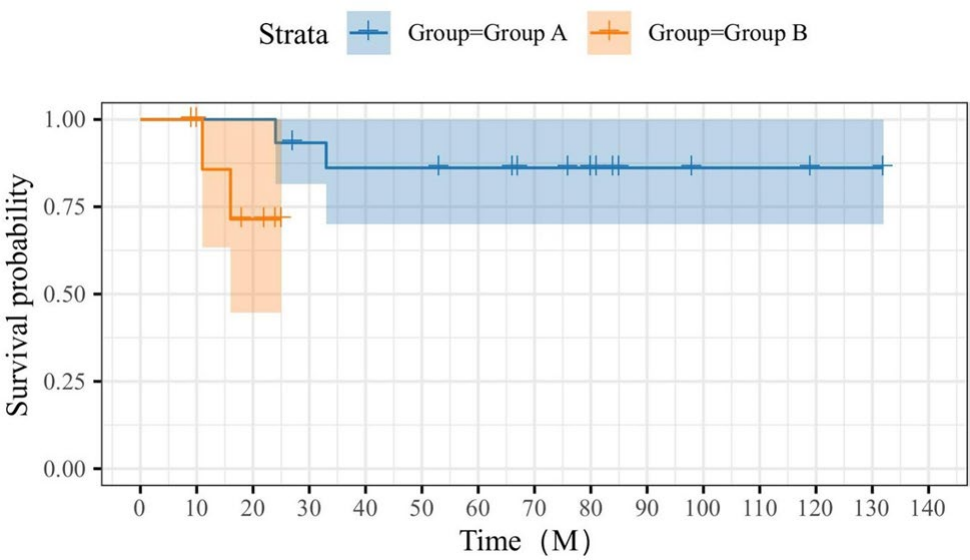
Survival curves.

See Fig. 10 for details.

#### Typical patient: 11 years after surgery

**Figure 11:**
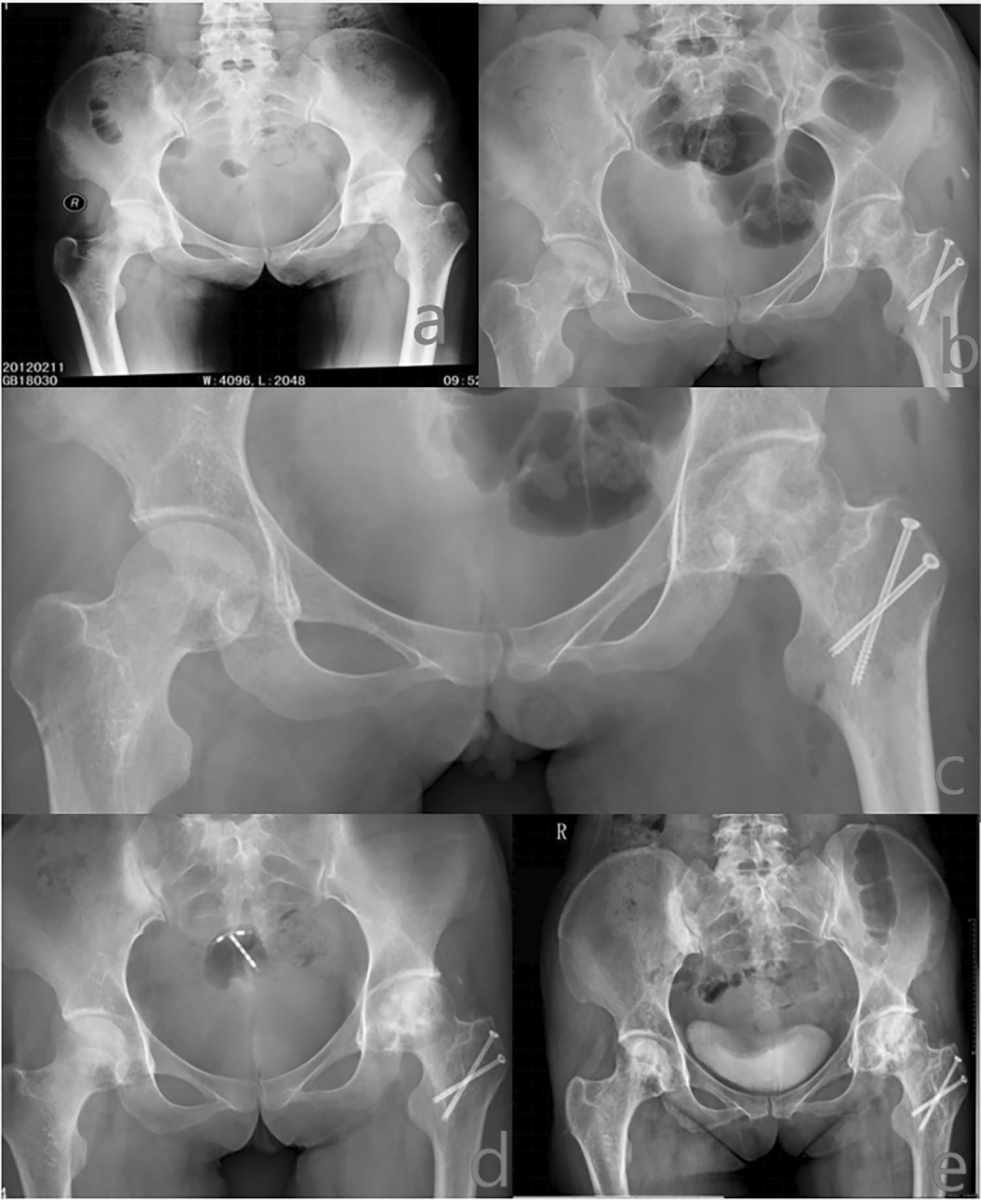
Preoperative, postoperative, 3 years postoperative, 7 years postoperative (femoral head shape is still good, no collapse), 11 years postoperative (femoral head shape is still good, no collapse): left femoral head has not collapsed, internal fixation at the greater trochanter is in place, and the compression implantation in the femoral head is healing well with the osteotomy at the greater trochanter.

**Figure 12:**
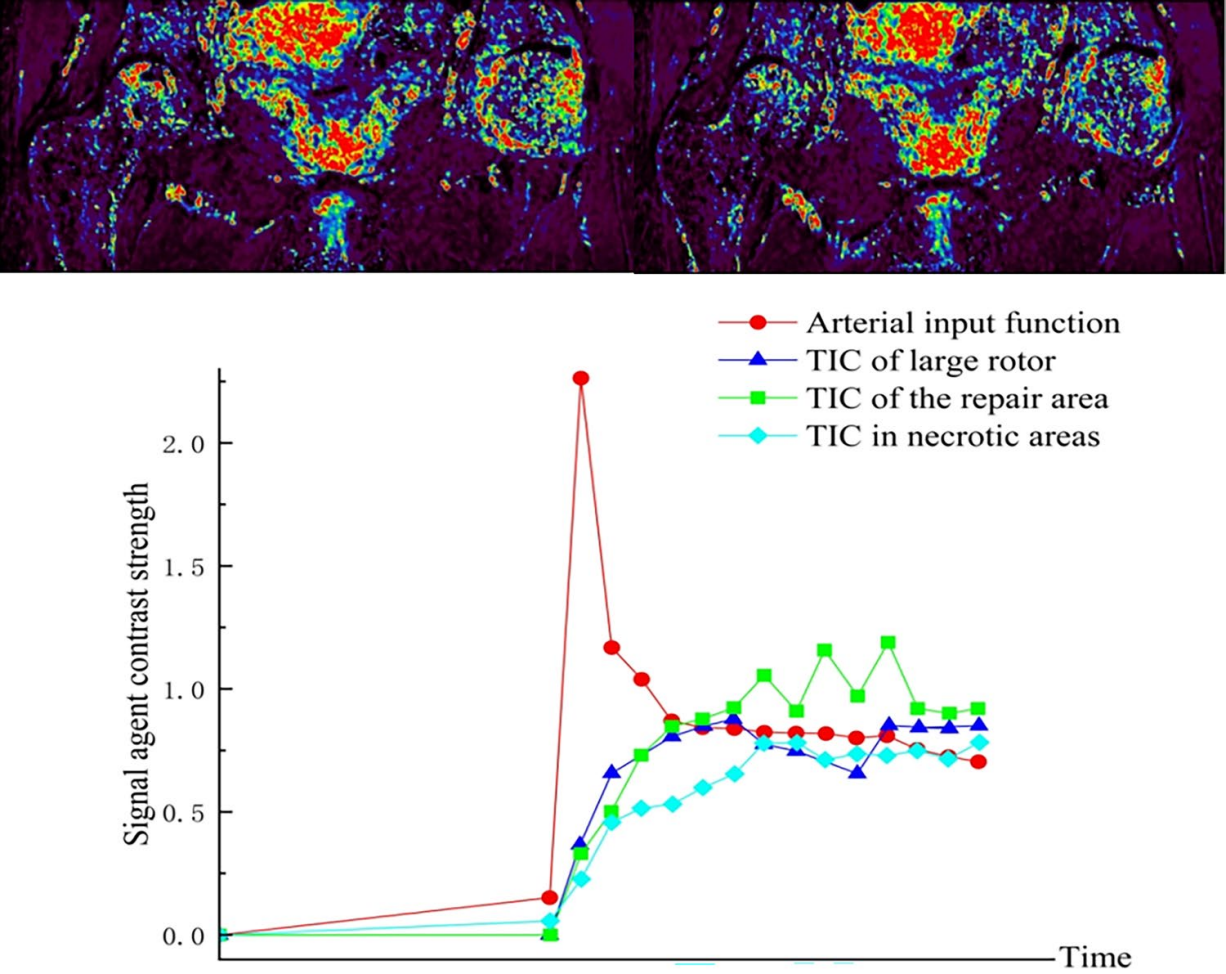
Eleven years after surgical dislocation, the blood perfusion curves of necrotic and repaired areas are close to the normal area, suggesting that the instability within the femoral head is effectively improved and blood perfusion is partially restored, with a better prognosis.

Patient: XX Hu, 30 years old, 11 years after SHD-IBG of the left hip. See Figs. 11 and 12 for details.

### Comprehensive analysis of patients with failed hip preservation

**Figure 13:**
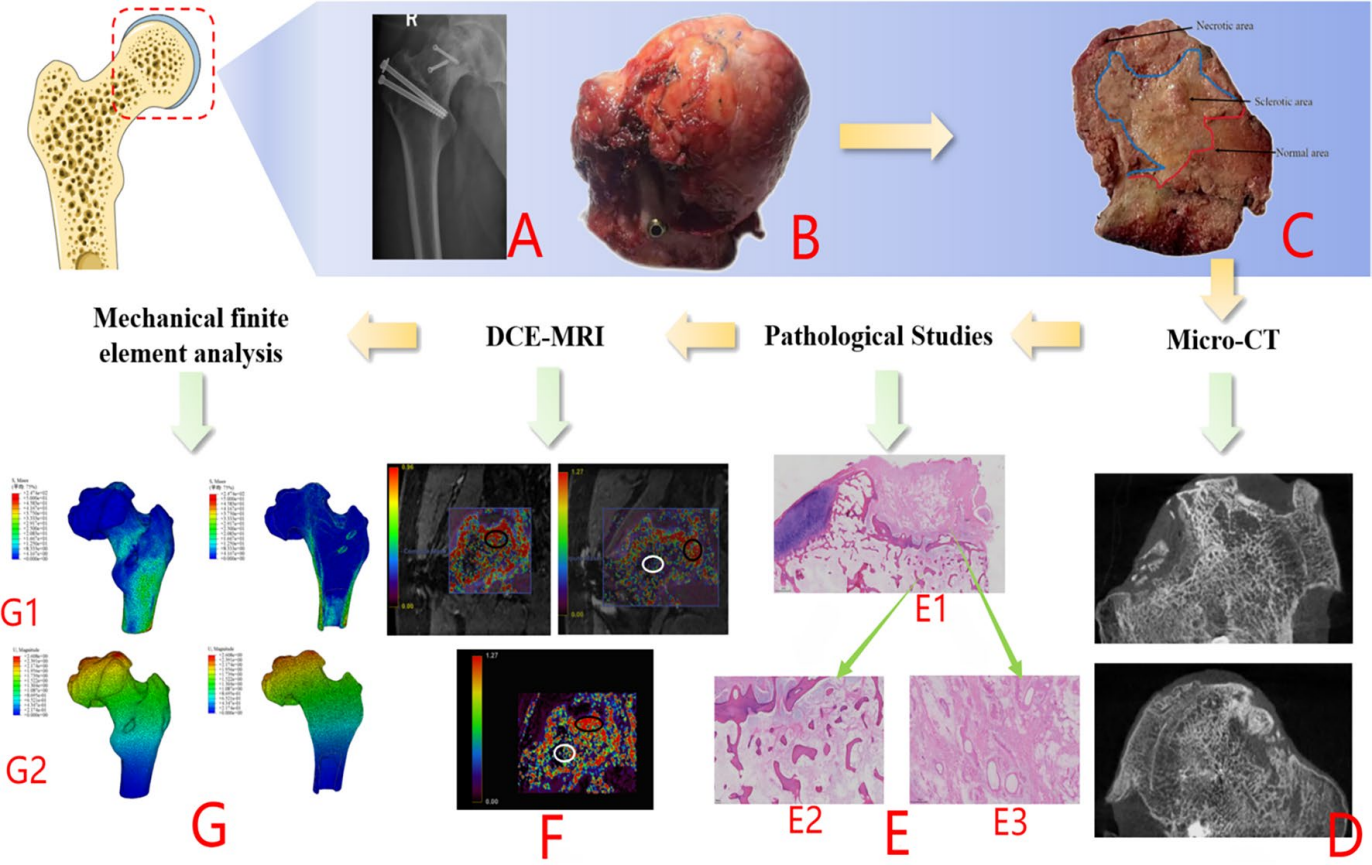
(**A**–**C**) The femoral head specimen shows obvious collapse, and the section shows separation of cartilage and subchondral bone and necrotic area, The bone in the necrotic area appears sediment-like, with the trabecular structure disappearing. Additionally, there is a sclerotic band, characterized by a high-density white band, mainly present in the area of normal bone tissue and the necrotic area. The trabeculae in this band are stout, and the trabecular space is narrow; (**D**) Micro-CT imaging of the femoral head confirms severe disruption of the internal microstructure, with the normal structure disappearing and the presence of fractures and stacking fusion; (**E**) HE staining of the coronal position of the femoral head shows the downward displacement of bone in the femoral head, with a large amount of fibrous connective tissue in the collapsed area, and the hyperplasia of blood vessels is clearly visible; a large number of newborn trabeculae can be seen in the sclerotic area, and the trabeculae are arranged tightly, with an increase in density and osteoclasts attached to the periphery. In the healthy area, the trabeculae are normally arranged and structurally intact; (**F**) (white ellipse ROI) poor perfusion, and abnormally enhanced perfusion in the area of the repair response (black ellipse ROI); (**G**) The maximum equivalent force of the stress (S-von-mises) (G1) was found in the cortical bone in the periphery of the femoral stem, and the equivalent force in the cortical bone on the upper and lower sides of the femoral neck was also relatively large. In the region of the femoral neck, the equivalent force values of the femoral model were similarly large. Conduction of stresses within the femoral head is visible in the section, but it is not continuous and does not form a continuous conduction structure. The maximum values of displacement (U-displacement) deformation (G2) were all seen in the femoral head region, with the peak displacement of the proximal femur located at the tip of the weight-bearing region of the femoral head, decreasing from proximal to distal.

Group A: Patient XX Zhu, right hip SONFH, failed hip preservation 24 months after SHD-IBG. See Fig. 13 for details.

**Figure 14:**
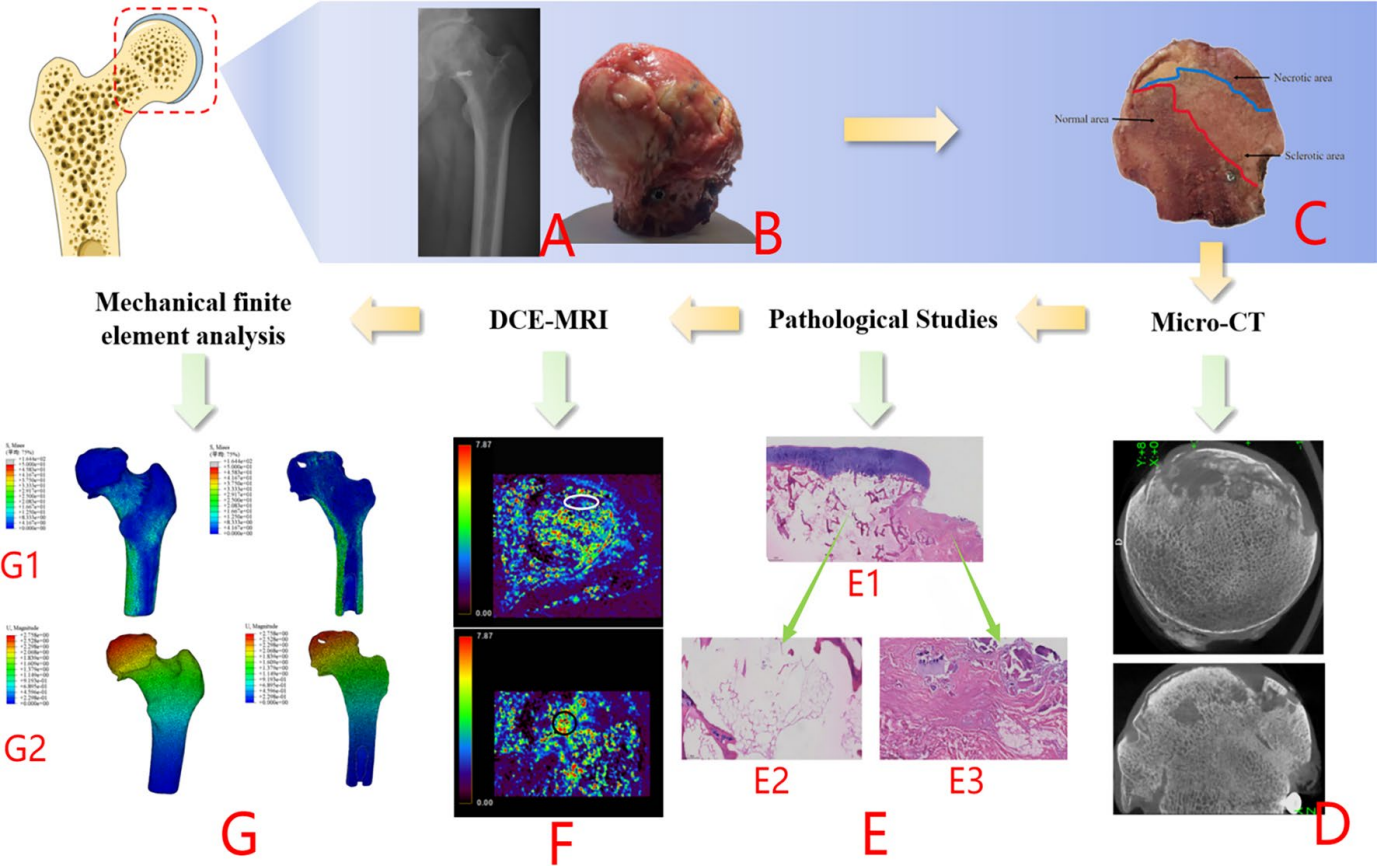
(**A**–**C**) The femoral head is collapsed and deformed, with local changes of articular cartilage and subchondral bone separation. The necrotic foci are demarcated from the surrounding tissues; (**D**) Micro-CT reveals that the bone trabeculae are thin and partially disordered in arrangement. The spacing of the bone trabeculae is wide, and some of the bone trabeculae are fractured. Additionally, the area of the collapsed femoral head surface is relatively small compared to that of patients in Group A. The surface of the femoral head collapsed, reducing its area. The trabecular structure was twisted, thinned, and broken. The subchondral bone was fractured, and the cartilage was separated from the subchondral bone; trabecular fractures were seen in the necrotic area, and the trabeculae in the subchondral bone area were thinned, with altered structural continuity, sparse breakage, and increased empty lacunae. In the subchondral bone area, the trabecular structure was scattered and missing; in the healthy area, the trabecular structure was intact and the thickness was evenly distributed; (**E**) HE staining of the femoral head coronal position showed that the trabeculae in the necrotic area were sparse, arranged haphazardly and without order, the continuity was lost, and the cells disappeared in the trabecular fossa; in the collapse area, the necrotic area was replaced by fibrous bone tissues. Osteoblasts disappeared in the bone trabecular fossa in the necrotic region and were accompanied by a variety of tissue proliferation, including connective tissue and neovascularisation; (**F**) (white ellipse ROI) poor perfusion and enhanced perfusion in the repair-responsive region (black ellipse ROI); (**G**) Stress (S-von-mises) Maximum equivalent force (G1) appeared in the region of the stress and the periphery of the femoral stem in the cortical bone; compared with the model of group A, the equivalent force in the femur model The equivalent force values of the femoral model were reduced at both the femoral neck and the lateral femoral stem cortex compared to the group A model. Stress transmission within the femoral head is also seen in the section. The maximum values of the displacement U-displacement deformation (G2) were found in the femoral head region, and the peak displacement of the proximal femoral osteotomy graft was located at the tip of the weight-bearing area of the femoral head, decreasing from proximal to distal.

Group B: Patient Yan X, left hip SONFH, failed hip preservation 16 months after PVIBGT. See Fig. 14 for details.

## Discussion

### Comparison of SHD-IBG and PVIBGT 1 year after operation

Comparison of the efficacy of the two groups at one year after surgery showed that the Harris scores of the hip joints of the patients in both the experimental and control groups were significantly improved at one year after surgery. X-ray, CT and MRI comparisons showed that the femoral heads of the patients in the two groups were in good shape, and the necrotic areas were well healed with the growth of new bone. The short-term follow-up results showed that PVIBGT was more effective than SHD-IBG in the treatment of ACRO IIIA femoral head necrosis.

The similarities between the two surgical procedures are: During the operation, necrotic bone and sclerotic bone were completely removed and autogenous cancellous bone was used to fill the necrotic area. The differences between the two surgical procedures are: (1) Different access: compared with PVIBGT, SHD facilitates better exposure of the femoral head, so it can remove the dead bone more thoroughly and is conducive to the observation of the blood supply of the femoral head; (2) Different indications: SHD-IBG is suitable for normal blood supply in the femoral head, and its main purpose is to restore the shape of the femoral head without destroying the microcirculation of the femoral head, while PVIBGT is mainly aimed at restoring the shape of the femoral head and improving the microcirculation of the femoral head at the same time. (3) The bone coverage at the fenestration is different: SHD-IBG will open a window in cortical bone in situ or use with muscular pedicle of the ilium flap coverage, while PVIBGT is rich blood supply of the ilium flap transferred to the femoral head in the window, provide enough mechanical support to maintain femoral head, on the basis of spherical shape, and can provide new blood for bone flap, to participate in the reconstruction of inducing osteoblast, conducive to the recovery of the subchondral bone. (4) Secondly, the sources of cancellous bone are different. SHD-IBG gathers autologous cancellous bone during the greater trochanteric osteotomy and the iliac, while PVIBGT obtains cancellous bone from the iliac bone. Whether such difference has any potential impact on the surgical efficacy needs further investigation^6^.

Therefore, the blood supply in the femoral head is the basis for choosing the two types of operation. We analysed the results of DCE-MRI images and time-intensity (TIC) curves of the two groups of patients separately: (1) In Group A, through the postoperative DCE-MRI images and TIC curves, it was found that the patient's affected side had uniform blood perfusion, the reinforcement curve of the repair area was close to the normal area without abnormal hyperperfusion, and blood perfusion existed in the necrotic area, which suggests that the femoral head is stable within the femoral head and has a good prognosis. The results showed that SHD-IBG did not damage the blood supply of the femoral head. () Through the preoperative, postoperative and postoperative follow-up of DCE-MRI images, TIC curves and quantitative changes of DCE-MRI in Group B, it can be found that PVIBGT improved the blood supply of the necrotic area of the femoral head and regulated the abnormal hyperperfusion of the repair area, with a lower risk of collapse and a better prognosis. It also provides new clinical research data to support that PVIBGT promotes the improvement of microcirculation in the femoral head.

### Medium and long-term outcome analysis of SHD-IBG

Since most of the patients who underwent hip-preserving surgery were younger than 40 years old, it is necessary to evaluate the postoperative outcomes of the patients through long-term follow-up. In the present study, a mid-to-long-term follow-up of 2.5–11 (an average of 77 months) years was performed for group A. We analyzed the survival rate and mean survival of patients who underwent this procedure. Only 2 patients in group A failing to preserve their hip and a THA conversion rate of 11.8% at 2.5–11 (an average of 77 months) years of follow-up. All of the remaining 13 had an improvement in their final Harris scores from preoperative levels, but 4 also showed a decrease in Harris scores that did not affect daily activities. The DCE-MRI at the final follow-up showed that the blood supply in the necrotic area of the femoral head of all patients was good, except 3 of them, who showed deterioration of the blood supply in the necrotic area, but they did not show obvious symptoms. It should be pointed out that they still need further medical care to prevent femoral head collapse.

### Analysis and summary of reasons for hip preservation failure

In this study, 3 of the 4 patients who failed were SONFH and the 3 SONFH patients still required continuous or intermittent oral hormonal medication after hip preservation.

First, we speculate that failure of hip preservation is related to continued glucocorticoid use^7^. Hormone-induced microenvironmental disorders within the femoral head, significant changes in the bone microstructure within the femoral head, changes in the thickness and density of trabeculae in the subchondral bone area and necrotic area, uneven thickness of trabeculae, haphazard arrangement, sparse fractures, trabecular fractures in multiple locations, disappearance of osteoblasts, significant increase in empty bone sockets, replacement of part of the bone tissue by fibrous tissue, and uneven distribution of bone density within the femoral head. The bone trabeculae in the sclerotic area were proliferated in large quantities, densely arranged, and the gap was narrowed. In the present study, postoperative ONFH specimens exhibited osteogenesis lagging behind the filling of broken bone and fibrous connective tissue. We believe that the reason for this phenomenon is the loss of the original framework of the osteogenic process after rapid osteoblast breakage and the rich blood supply during the destructive repair process, as shown by the DCE-MRI that there was an abnormal hyperperfusion in the femoral head of both A and B patients^8^.

Secondly, both procedures require attention to the issues of intraoperative dead bone clean-up, over-implantation of compression and postoperative length and extent of cartilage and implanted bone. As the maximum equivalent force in the mechanical analysis of both patients A and B appeared in the cortical bone at the periphery of the femoral stem, the conduction of internal femoral head stresses was visible in the section, but a continuous conduction structure was not formed^9^.

Third, in this study, there was a problem of bone resorption between the implanted bone and the surrounding normal bone. In some patients, the anterolateral weight-bearing area of the femoral head was found to be extensively necrotic before surgery, and the dead bone in the head was close to the subchondral bone. Although the dead bone was thoroughly cleaned up during the operation, the long-term follow-up found that the subchondral implanted bone and the cartilage could not be integrated together, and the cartilage and the subchondral bone were separated from the cartilage^10^.

In addition, through A comprehensive comparison of the two groups of patients with hip preservation failure, we found that the collapse area of the lateral column in group A was larger than that in group B, the collapse was more obvious, the trabecular bone structure disappeared more than that in group B, and the abnormal hyperperfusion in the necrotic area was obvious.

### Limitations and prospects

However, the limitations of this study must be acknowledged. First, the postoperative follow-up time of PVIBGT was short, only one year, and further follow-up is still needed; second, a total of 30 samples were selected for the study, which is a small sample size. Third, the blood supply of the femoral head analysed by DCE-MRI is susceptible to the influence of parameter settings, which cannot guarantee the accuracy and is different from that seen in the operation.

In the future, we will follow up the existing patients for a longer period of time and include more patients in the study in a more rigorous manner. In addition, we will further investigate the long-term effects of the femoral head blood supply of both surgical operations in combination with DCE-MRI.

## Conclusion

Firstly, by comparing the efficacy of SHD-IBG and PVIBGT at 1 year after surgery, we found that the short-term efficacy of PVIBGT was better than that of SHD-IBG. Second, SHD-IBG has been shown to delay the progression of osteonecrosis of the femoral head with a long-term follow-up of 2.5 to 11 months (mean 77 months). Finally, combined with the comprehensive analysis of the two groups of patients with hip preservation failure, it was shown that whether the lateral column further collapsed had a significant impact on the outcome of femoral head necrosis, and attention should be paid to the biomechanical support of the lateral column during the operation. In addition, DCE-MRI can clearly show the changes of blood supply in the femoral head, which can objectively show the effect of hip preservation surgery.

## Data Availability

The datasets used during the current study are available from the corresponding author on reasonable request.
